# Mesenchymal Stem Cell Conditioned Media Ameliorate Psoriasis Vulgaris: A Case Study

**DOI:** 10.1155/2019/8309103

**Published:** 2019-05-02

**Authors:** Rajasekar Seetharaman, Anjum Mahmood, Prashant Kshatriya, Divyang Patel, Anand Srivastava

**Affiliations:** ^1^GIOSTAR Research Inc. Pvt. Ltd., Ahmedabad, Gujrat, India; ^2^Global Institute of Stem Cell Therapy and Research, 4660 La Jolla Village Drive, San Diego, CA 92122, USA

## Abstract

Psoriasis, an autoimmune disease, affects a vast number of peoples around the world. In this report, we discuss our findings about a scalp psoriasis suffering patient with a Psoriasis Scalp Severity Index (PSSI) score of 28, who was treated with Mesenchymal stem cell conditioned media (MSC-CM). Remarkably, complete regression was recorded within a treatment period of one month only (PSSI score of 0). A number of bioactive factors like cytokines and growth factors secreted by MSCs in the conditioned medium are very likely to be the principle molecules which play a vital role in skin regeneration. Treatment using MSC-CM appears to be an effective tool for tackling the psoriatic problem and, thus, may offer a new avenue of therapy which could be considered as an alternative approach to overcome the limitations of the cell-based therapy.

## 1. Introduction

Psoriasis is a chronic disease thought to be of autoimmune origin which is characterized by patches on the skin and nails. It has been considered as a serious skin related problem affecting approximately 100 million individuals worldwide. About 2% of the world population and 0.44-2.8% of the Indian population were affected by psoriasis in 2016-2017 [1, 2]. Plaque, guttate, inverse, pustular, and erythrodermic are the five major types of psoriasis. Plaque psoriasis, also known as psoriasis vulgaris, is the most common form of the disease (about 90% of the cases) [3] which typically presents with red patches with white scales on top. Psoriasis vulgaris which commonly affects the areas includes scalp, knees, elbows, hands, nails, and feet [4].

Psoriasis, an autoimmune-inflammatory disease probably predisposed due to genetic makeup, is mediated by T-helper cells. Polymorphism, referred to as differences in DNA sequences of a gene, can be incurred by various external agents like chemicals, viruses, or radiation. Polymorphisms in genes of Th2 cytokine/regulatory T-cell (interleukin-10/IL10), Th1/Th17 cytokine (IL-12B and IL-23R), and tumour necrosis factor alpha (TNFAIP3; TNIP1) confer which increased other risks like cardiovascular diseases amongst psoriasis patients [5–7]. Single nucleotide alteration caused polymorphism in Th1 proinflammatory cytokine gene IL-2 [–330 (G/T)] which has been shown to be associated with greater disease severity in the Indian population [1]. On the other hand, another gene polymorphism occurring in Th-2 cytokine/regulatory T-cell (IL-4) has been shown to be protective against psoriasis [5]. Upregulation in the levels of inflammatory cytokines leads to psoriasis which also can be associated with an increased risk of psoriatic arthritis, lymphomas, cardiovascular risk, Crohn's disease, and depression [3]. There is no permanent cure for psoriasis, though steroid creams, vitamin D3 cream, ultraviolet light, and immune system suppressing medications (methotrexate) have been in wide use to help control the symptoms with some success [8, 9].

Mesenchymal stem cells (MSCs) are multipotent adult stem cells which have an excellent capacity to proliferate for an extended period of time while maintaining the undifferentiated cell status. The resulting daughter cells can differentiate into various types of cells of host tissues and thus help repair wear and tear incurred [10]. MSCs have a potential to serve as a powerful tool in cell-based therapy due to their tissue regenerative and host immune modulatory capabilities. The functions exhibited by MSCs have attracted a number of scientists and clinicians to investigate the mechanisms involved in their curative and tissue regeneration functions. A very few articles have reported the effectiveness of stromal vascular fraction (SVF)/MSC therapy in curing psoriasis by regulating the immune systems. Lee et al. [11] reported that human umbilical cord blood-derived mesenchymal stem cells (hUCB-MSCs) ameliorate psoriasis-like skin inflammation in mice and have regulatory effects on immune cells including CD4^+^ T cells and dendritic cells. The first case study on intravenous infusion of SVF into psoriasis patient demonstrated a significant decrease in symptoms with a noticeable difference in skin appearance, psoriasis area, and severity index (PASI) score reduction (from 50.4 to 0.3) [12]. Chen* et al*. [13] reported that umbilical cord-derived MSC (UC-MSC) infusion effectively reduced psoriasis in human subjects. It was believed that MSCs' migration into the skin lesions and their immunomodulatory, autoimmune inhibitory, and paracrine effects were the principal factors behind the ameliorative effects. Other recent preclinical studies have shown that stem cell-derived conditioned media (CM) exhibit effective healing of psoriasis-like wounds and thus CM is an alternative for several cell-based therapies [14, 15]. The paracrine factors including growth factors, chemokines, and cytokines secreted from stem cells play a major role in wound healing [16] and these molecules are present in CM or spent medium harvested from cultured cells [17]. In short, CM can serve as a novel treatment approach in regenerative medicine which has been shown to have a successful outcome in preclinical studies. However, a very few reports are available on the clinical application of CM for treatment of any disease. Based on the principles and importance of CM, the present study was aimed at investigating the effect of MSC-CM on a patient suffering from psoriasis. This study is believed to be the first clinical report on the use of MSC-CM to treat psoriasis.

## 2. Case Report

### 2.1. Patient

A 38-year-old male patient, who was suffering from psoriasis vulgaris for 2 years, paid a visit to our centre. Preliminary examination of the patient showed that numerous erythematous plaques with numerous silvery scales present all over the scalp including the area behind the ears. The severity of the disease was assessed to be 28 on Psoriasis Scalp Severity Index (PSSI), calculated by the standard method which combines the severity (erythema, induration, and desquamation) and percentage of affected area.

### 2.2. Preparation of MSC-Conditioned Media

**Figure 1:**
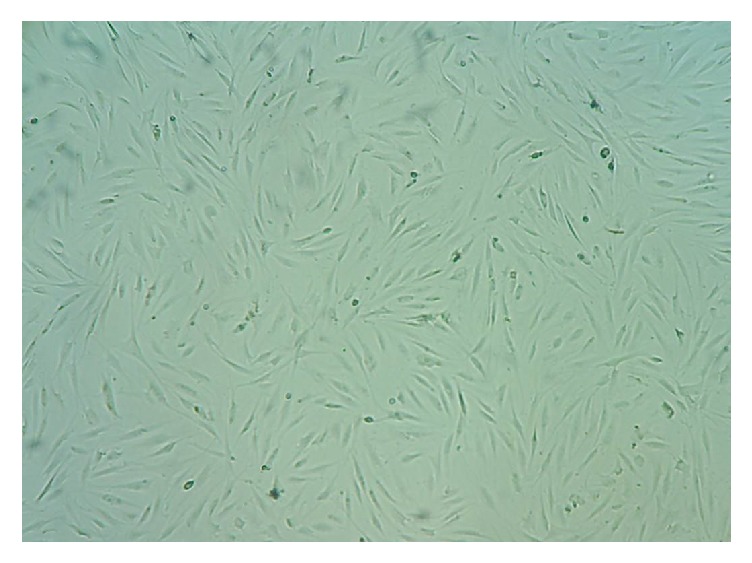
Phase-contrast microscopic image showing spindle shaped MSCs (× 100).

Adipose tissue was collected from a healthy volunteer by lipoaspiration by a plastic surgeon under the aseptic conditions in the O.T. About 100 ml of fat was aspirated out from the waist area and collected in a sterile container. The fat tissue contacting stem cells was processed in a biosafety laminar airflow chamber. MSCs were isolated from adipose tissue by standard enzymatic digestion method with 0.1% collagenase type I. Following the centrifugation, the resulting pellet was cultured in DMEM medium (Invitrogen, Paisley UK) supplemented with 10% foetal bovine serum (FBS) and 1% penicillin/streptomycin, at 37°C in humidified atmosphere containing 5% CO_2._ The media were changed after every 3 days. About 5×10^6^ MSCs of passage 2 were seeded in each T175 culture flask (n=10) containing 30 ml of DMEM medium supplemented with 10% FBS. MSCs were confirmed with spindle shaped morphology and free from any contamination (Figure 1) using a phase-contrast microscope. When cells attained 90% confluence at passage 2, the culture media were replaced with serum-free DMEM. After 72 h of incubation, resulting MSC-CM was collected, centrifuged at 2000 rpm for 5 min to remove the cell debris, filtered through 0.22-*μ*m filter, and then concentrated (10 times) by ultrafiltration using centrifugal filtering units with a cut-off value of 3 kDa (Amicon Ultra-15; Millipore, MA), according to the manufacturer's instructions. The concentrated MSC-CM was aliquoted and stored at -20°C until use. MSC-CM was topically applied on the afflicted areas once a day over a period of one month. Clinical parameters like severity, changes, and clearance of psoriatic plaques were monitored at regular intervals.

### 2.3. MSC-CM Ameliorates Psoriasis Vulgaris

**Figure 2:**
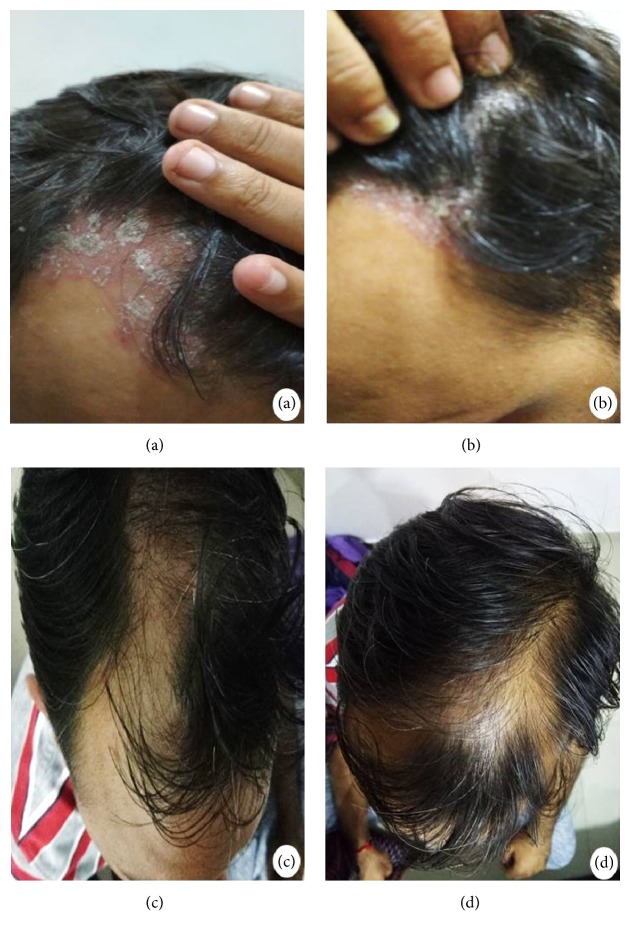
*Effect of MSC-CM on psoriasis vulgaris*. The scalp of patient showing numerous erythematous plaques with adherent silvery flakes before MSC-CM-treatment (a, b). Regression of psoriasis and a complete clearance of inflammatory erythematous plaques recorded after topical application of MSC-CM for a period of one month (c, d).

Numerous psoriatic erythematous plaques with adhering silvery scales over the different regions of the scalp were observed before the treatment regimen started. In general, the number of the scales declined significantly within 2 weeks of topical application of MSC-CM. Interestingly, clearance of silvery scales and severity of psoriatic plaques were completely abolished within one month of the treatment (Figure 2). The PSSI score reduced to 0 from 28 and regression of the disease continued for 6 months of follow-up. The patient did not take any other medication during the follow-up period of six months and led an improved quality of life without any adverse side effects.

## 3. Discussion

Psoriasis is an autoimmune disease mediated by hyperactivity of T-helper cells. Increases in the levels of inflammatory cytokines triggered by T cells lead to psoriasis and other associated diseases [3]. Therapies using multipotent MSCs have been shown to be effective in treating psoriasis and psoriasis-like other skin diseases. Clinical benefits may be attributed to MSCs engraftment or to their paracrine/immunomodulatory effects. However, transplanting MSCs come with few limitations like low survivability of cells in the host due to harsh microenvironment and cell loss because of poor or no cell adhesion [18]. Hence there is need of the hour to find an alternative for cell-based therapy.

Cell-free products of MSC origin are effective in wound healing and skin diseases. In this study, we demonstrated that MSC-CM can be used to treat patients with chronic psoriasis. Prior to MSC-CM treatment, the patient had received different medications but without any noticeable effective outcome. Remarkably, topical application of MSC-CM for a period of only one month completely abolished the erythematous plaques and resulted into a complete clearance of adherent silvery scales over the scalp. Further, the severity of psoriasis was completely reduced, from PSSI score of 28 to 0. This regeneration of tissue and improvement of qualitative appearance skin may be mediated by the growth factors, chemokines, and cytokines present in MSC-CM. Previous reports have shown that the paracrine factors secreted by MSC present in CM play a vital role in the healing of psoriasis-like wounds [16, 17]. Kim et al. [19] stated that adipose-derived stem cell (ADSC)-CM has regenerative effects on skin wounds. It stimulates both collagen synthesis, migration of dermal fibroblasts and promotes wound healing in animal models. ADSC-CM also upregulates the transcription of type I procollagen-alpha-1 chain gene of fibroblasts and induces Rho-associated kinase (RhoA-ROCK) signalling pathway, which leads to the proliferation of keratinocytes and dermal fibroblasts. In other studies [20, 21], MSC-CM promoted the recovery of skin burn wounds in rats, marked by an acceleration of wound closure, greater numbers of fibroblasts around and injured tissue and blood vessels, high epithelialization ratio, and high density of collagen fibres. It was suggested that basic fibroblast growth factor (bFGF) played an important role in the tissues regeneration of skin burn treated by MSC-CM.

*In vitro* and* in vivo* studies involving UC-MSC-CM demonstrated that its application caused an increase in the proliferation and migration of dermal fibroblasts, decrease in the ratio of transforming growth factor-*β*1/*β*3, and an increase in the ratio of matrix metalloproteinase over counter agent tissue inhibitor of metalloproteinases [22]. Similarly, human embryonic stem cell (hESC)-derived endothelial precursor cells CM is a rich source of a number of growth factors like epidermal growth factor (EGF), bFGF, fractalkine, granulocyte-macrophage colony-stimulating factor (GM-CSF), and interleukin (IL)-6. It was successfully used in the treatment of excisional wound healing in rats [23]. A comparative study revealed that wound healing by bone-marrow derived mesenchymal stem cell (BMMSC)-CM was significantly higher than that by fibroblast-CM [16]. The fact that BMMSC-CM had higher levels of paracrine factors than those in fibroblast-CM indicated the importance of origin of cells played a significant role in the production of paracrine factors. Other growth factors like Vascular endothelial growth factor (VEGF), insulin like growth factor (IGF), EGF, keratinocyte growth factor (KGF), angioprotein-1 (Ang-1), stromal derived factor-1, and erythropoietin (EPO) were also present in BMMSC-CM. With the supporting evidence of previous reports, the ameliorative effect of MSC-CM exhibited in this present study could be attributed to the presence of numerous growth factors secreted by MSCs in the media.

## 4. Conclusions

This is the first case report which demonstrates the ameliorative effect of MSC-CM on psoriasis vulgaris. MSC-CM is likely to have a wide range of cytokines and growth factors which can directly act on resident skin cells and thus can help in the skin regeneration. The active bioactive ingredients and their needed combination are yet to be determined. Use of MSC-CM instead of direct implantation of MSCs to tackle the issue offers an alternative approach which overcomes a number of limitations of cell-based therapy. In conclusion, treatment using MSC-CM appears to be highly effective for the treatment of psoriasis and may represent a new avenue of therapy. Further investigations for addressing a number of question provoked by the findings reported here, such as long-term effects (over a period of years), induced changes at cellular and histological levels, and identification of involved bioactive molecules, demand more studies.
